# A Translational Model for Repeated Episodes of Joint Inflammation: Welfare, Clinical and Synovial Fluid Biomarker Assessment

**DOI:** 10.3390/ani13203190

**Published:** 2023-10-12

**Authors:** Clodagh M. Kearney, Nicoline M. Korthagen, Saskia G. M. Plomp, Margot C. Labberté, Janny C. de Grauw, P. René van Weeren, Pieter A. J. Brama

**Affiliations:** 1 UCD School of Veterinary Medicine, University College Dublin, D04 W6F6 Dublin, Irelandpieter.brama@ucd.ie (P.A.J.B.); 2 Department of Clinical Sciences, Faculty of Veterinary Medicine, Utrecht University, 3584 CM Utrecht, The Netherlandss.g.m.plomp@uu.nl (S.G.M.P.); r.vanweeren@uu.nl (P.R.v.W.); 3 Department of Clinical Sciences and Services, Royal Veterinary College, University of London, Hatfield AL9 7TA, UK

**Keywords:** inflammation, recurrent, joint, lipopolysaccharide, animal model, horse

## Abstract

**Simple Summary:**

Repeated episodes of joint inflammation play a key role in the progression of joint diseases such as osteoarthritis. In order to better understand diseases and develop treatments, animal studies are needed. Most models of joint inflammation cause severe discomfort and irreversible damage to joints which is neither truly reflective of naturally occurring disease processes nor desirable for the welfare of the experimental animals. This study examines a potential model of recurrent lower levels of inflammation. Minute doses of lipopolysaccharide (LPS), a substance that causes inflammation, were injected into the joints of horses three times at two-week intervals, and the effect of these injections on the horses’ comfort and welfare and markers of inflammation within the joint fluid were closely monitored. We found that each of these injections produced reliable and comparable levels of inflammation within the joints, with minimal impact on the horses’ comfort and welfare. The joints also showed complete recovery when re-examined at a later timepoint. These results suggest that this model has potential as a refined translational model of repeated episodes of joint inflammation that is more representative of natural disease states and can be used to evaluate potential therapeutics over several weeks.

**Abstract:**

This study investigates repeated low-dose lipopolysaccharide (LPS) injections in equine joints as a model for recurrent joint inflammation and its impact on animal welfare. Joint inflammation was induced in eight horses by injecting 0.25 ng of LPS three times at two-week intervals. Welfare scores and clinical parameters were recorded at baseline and over 168 h post-injection. Serial synoviocentesis was performed for the analysis of a panel of synovial fluid biomarkers of inflammation and cartilage turnover. Clinical parameters and a final synoviocentesis were also performed eight weeks after the last sampling point to assess the recovery of normal joint homeostasis. Statistical methods were used to compare the magnitude of response to each of the 3 LPS inductions and to compare the baseline and final measurements. Each LPS injection produced consistent clinical and biomarker responses, with minimal changes in welfare scores. General matrix metalloproteinase (MMP) activity and joint circumference showed greater response to the second LPS induction, but response to the third was comparable to the first. Gylcosaminoglycans (GAG) levels showed a significantly decreased response with each induction, while collagen-cleavage neoepitope of type II collagen (C2C) and carboxypropetide of type II collagen epitope (CPII) showed quicker responses to the second and third inductions. All parameters were comparable to baseline values at the final timepoint. In conclusion, a consistent, reliable intra-articular inflammatory response can be achieved with repeated injections of 0.25 ng LPS, with minimal impact on animal welfare, suggesting potential as a refined translational model of recurrent joint inflammation.

## 1. Introduction

Osteoarthritis (OA) is one of the leading causes of disability worldwide, impacting quality of life through pain and loss of mobility, in addition to having considerable economic impacts [1]. OA is also a major health issue for the horse, being recognized as the single greatest cause of economic loss in the equine industry [2]. While previously OA was considered a disease of “wear and tear” and research was focused on late-stage structural changes to cartilage and bone, recurrent and sustained inflammatory processes within the joint are now recognized as essential in the pathogenesis and progression of the disease [3]. At present, there is no truly disease-modifying treatment for OA in either horses or humans. The currently commonly available therapies are symptomatic, show limited effectiveness, and come with adverse effects [4,5]. Many novel therapeutics, ranging from oral neutraceuticals [6,7] to gene therapies [8], have been investigated in the drive to develop better therapies for controlling joint inflammation and therefore preventing OA development and progression in both horses and humans.

In addition to potentially benefiting from any therapeutics developed, the horse has been recognized as a suitable translational model for joint research [9,10,11,12,13]. The size of the horse allows for successful repeated sampling of synovial fluid [9], meaning that joint health status can be monitored over time and changes in synovial fluid composition provide valuable real-time insight into both healthy [14,15,16,17] and diseased joints [18,19,20]. In both humans and equines, a large number of synovial and serum biomarkers have been investigated and validated as measures of joint health or disease [18,21], providing a panel of markers that can be used to characterize and quantify joint inflammation. 

First used by Firth in the 1980s in a model to study infectious arthritis [22], intra-articular lipopolysaccharide (LPS) has been found to elicit marked joint inflammation in horses. Since then, variations of this reversible, transient equine inflammation model have been used to study clinical signs of inflammation, pathways of inflammation, and the effects of various therapeutics [23,24,25,26,27]. In addition, in line with welfare considerations and the 3Rs in animal models, refinements reducing LPS dosage to sub-nano dosages have demonstrated that a reliable inflammatory response could still be induced with a dose as low as 0.25 ng [24,28]. 

Nevertheless, the transient nature of the induced inflammatory response could be regarded as a limitation for its use in assessing therapeutics for recurrent inflammation, which is considered to be the hallmark of OA development. To overcome this problem, Cokelaere et al. [29] further expanded the model by repeating LPS challenges in a sequential fashion to simulate repeated inflammatory flares. However, they reported that the intra-articular inflammatory response was inconsistent and less marked after the second and third inductions, suggesting tolerance with repeated exposure to LPS. We recently also used repeated inductions with LPS to investigate the treatment effects of intra-articular corticosteroids [30] and concluded contrarily, that consistent responses were found with each induction. Given the discrepancy in these findings, the aim of the present study was to further interrogate the changes in welfare, clinical, and synovial fluid biomarkers in response to repeated inductions of inflammation with low doses of LPS. The second aim of this study was to investigate if this repeated inflammation model has potential long-term consequences for the joints used. To do this, we analyzed data and samples collected at later timepoints from control joints used in previous studies. We hypothesize that repeated intra-articular injections of 0.25 ng of LPS produce a consistent and equivocal level of inflammation and would not lead to any persistent changes or significant welfare concerns. Further understanding of the inflammatory responses with repeated inflammation in this model would strengthen the model as a valid translational preclinical model for testing novel therapeutics for OA in humans and horses.

## 2. Materials and Methods

### 2.1. Study Design

**Figure 1:**
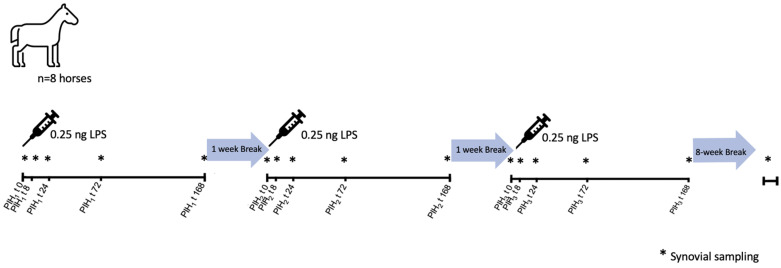
Timeline LPS inductions and synovial fluid (SF) sampling middle carpal joint of 8 horses. Post Induction Hour (PIH) is the time (t) in hours following each joint injection (1, 2 or 3) with lipopolysaccharide (LPS).

The data presented here were obtained as part of a larger investigation into the effects of several intra-articular therapeutics in a bilateral LPS model. For the presented study we further evaluated control-treated joints to characterize the repeated LPS induction model in detail. A timeline of the study is illustrated in Figure 1.

### 2.2. Experimental Animals 

Eight horses with no known history of forelimb musculoskeletal problems were selected from the University research herd for use in this experiment. The horses were of mixed breeds, age, gender, and size (6 mares and 2 geldings; age 14.6 ± 2.4 years (mean +/− SD), bodyweight 370.4 ± 27.6 kg (mean +/− SD). Each horse was examined by experienced clinicians prior to the start of the study to exclude any clinical evidence of joint inflammation, lameness, or radiographic abnormalities of the carpal joints. During the experiment, the horses were kept on wood shaving bedding in individual stables in a familiar environment. They were fed concentrates once daily, regular hay was provided, and water was provided ad libitum. During the break weeks between the LPS inductions and subsequent sampling periods, the horses were turned out on grass pasture in a familiar group. During these weeks, health checks were performed once daily by an experienced equine attendant who monitored the demeanor, appetite, and mobility of each horse.

### 2.3. Induction of Inflammation 

LPS from *Escherichia coli* O55:B5 (catalogue number L5418; Sigma-Aldrich Ireland Ltd., Arklow, Co., Wicklow, Ireland) was diluted to a final concentration of 0.25 ng/mL in sterile lactated Ringer’s solution. Horses were sedated with a combination of xylazine (0.2–0.5 mg/kg, Chanazine 10%^®^; Chanelle, Galway, Ireland) and butorphanol (0.01–0.02 mg/kg; Alvegesic vet 10^®^, ALVETRA u. WERFFT GmbH, Vienna, Austria) administered intravenously, and both dorsal carpal regions of each horse were clipped and just prior to arthrocentesis were scrubbed with gauze swabs soaked in a dilute chlorhexidine solution for a minimum 5 min contact time, and then rinsed with a 70% alcohol solution. At post-injection hour (PIH) t0 arthrocentesis was performed with a 20 G × 40 mm needle and 1 mL LPS solution was delivered aseptically into each middle carpal joint after withdrawal of a 4–5 mL synovial fluid (SF) sample. For each arthrocentesis or joint injection, the limb was held with the carpus partially flexed. The needle was placed just medial to the extensor carpi radialis muscle midway between the distal border of the radial carpal bone and the medial aspect of the proximal third carpal bone.

### 2.4. Welfare Monitoring 

Comprehensive clinical exams and welfare assessments were performed on each animal before the initial inductions of inflammation and then every 2 h over the subsequent 8 h. Following this, the same checks were performed once daily throughout the sampling weeks until PIH t168. For each of these timepoints, a composite welfare score (CWS) was assigned. The CWS is the aggregate of scores (each on a scale of 0–4) in 4 categories: food and water intake; clinical parameters (temperature, pulse, and respiratory rate); natural behaviour; and provoked behaviour. This scoring system has been designed and implemented by our group for bilateral equine LPS model studies to monitor welfare and fulfil institutional and national ethical regulatory requirements (Appendix A).

### 2.5. Clinical Assessment of Joint Inflammation

At each of the timepoints described above, prior to any procedure, effusion of the middle carpal joint was graded on a 0–4 scale, as previously described [31]. Joint circumference was measured at the level of the accessory carpal bone with a tape measure. Joint flexion was graded on a 0–4 scale, as a measure of the horses’ tolerance of passive flexion of the joint. All scores were assigned and recorded by the same experienced clinician.

### 2.6. Synovial Fluid Analysis

At fixed time points after each LPS induction (PIH t0, t8, t24, t72, and t168), arthrocentesis of each joint was performed under sedation as previously described, with a 4–5 mL sample being collected each time. A final arthrocentesis was also performed at a later timepoint not associated with an induction of LPS, 8 weeks after the PIH3 t168 sampling (Figure 1). A portion of the synovial fluid was separated for evaluation of manual white blood cell count (WBC) and total protein (TP) measurement (refractometer). The remainder was immediately centrifuged in plain tubes for 15 min at 4 °C at 10,000 rpm, and then aliquoted and stored at −80 °C until further analysis. The timeline of synovial fluid sampling for the experiment is illustrated in Figure 1.

### 2.7. Synovial Fluid Biomarker Analysis

A total of eight assays were performed on each synovial fluid sample, apart from the final timepoint for which bradykinin was not measured. 

Prostaglandin E2 (PGE2) concentrations were measured using high-performance liquid chromatography (HPLC)–tandem mass spectrometry (MS/MS) analysis as described previously [32]. 

C–C motif chemokine ligand 2 (CCL2) and tumor necrosis factor-alfa (TNF-alfa) concentrations were quantified using commercial equine-specific ELISA kits (DIY0694E-003 Kingfisher Biotech, St. Paul, MN, USA and #ESS0017, Thermo Fisher Scientific, Waltham, MA, USA) using an adapted protocol as previously described [30].

General matrix metalloproteinase (MMP) activity was measured using cleavage of fluorogenic substrate FS-6 (Calbiochem, San Diego, CA, USA) as previously described [29]. 

Glycosaminoglycan (GAG) concentrations were measured using a modified 1,9-dimethylmethyleneblue assay adapted for use in microtiter plates, as previously described [25].

Commercial ELISA kits were used to determine concentrations of collagen-cleavage neoepitope of type II collagen (C2C), carboxypropeptide of type II collagen epitope (CPII) (IBEX Technologies, Mont-Royal, QC, Canada) and Bradykinin (Peninsula Laboratories, San Carlos, CA, USA) in accordance with the manufacturer’s recommendations. 

### 2.8. Statistical Analysis

An a priori power analysis was performed to determine the number of animals that should be used. The power calculation based on the differences in synovial fluid biomarkers found in earlier studies using the LPS model [25,27,33] suggested that 8 horses would give a power of 0.8 and an alpha error rate of 0.05. Data are presented as the mean ± standard deviation (SD), except for the clinical scores that used ordinal scales for which the mode was presented (composite welfare scores, joint effusion, joint circumference, and joint flexion). 

For the first research question investigating if there is a difference in response to LPS with repeat inductions, mixed effects models were fit for each measure against time of peak as a categorical variable with horse ID as a random effect. Significance was set at *p* < 0.05 for all statistical analyses (*p* < 0.0045 with Bonferroni correction for 11 variables: carpal circumference & 10 synovial biomarkers).

For the second research question, looking at the difference between the baseline measurements and the measurements at the final timepoint, paired *t*-tests were used, except when inspection of the differences in circumference between the timepoints indicated non-normality, in which instances the Wilcoxon signed rank test was used as a non-parametric alternative to the paired *t*-test. Significance was set at *p* < 0.05 for all statistical analyses (*p* < 0.005 with Bonferroni correction for 10 variables: carpal circumference and 9 synovial biomarkers). Statistical analyses were performed using Stata Statistical Software: Release 15 (StataCorp LLC, College Station, TX, USA).

## 3. Results

In the third phase of the study, the results for only seven horses are reported, as one horse sustained a hind limb injury at pasture during the break period and, therefore, did not undergo the third induction with LPS. 

### 3.1. Research Question 1: Is There Evidence of Difference in Response to LPS with Repeat Inductions?

**Figure 2:**
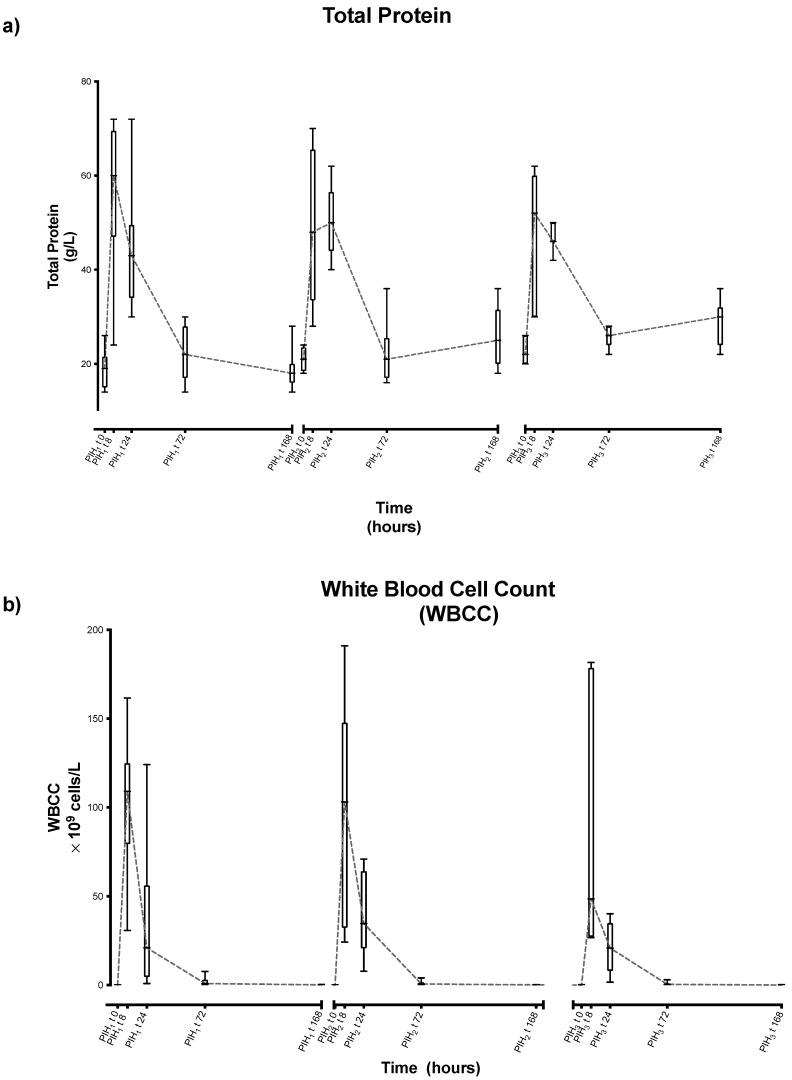
Synovial fluid (**a**) total protein and (**b**) white blood cell count (WBCC) over time following repeated induction of inflammation with intra-articular injections of 0.25 ng of LPS in the middle carpal joint of horses at PIH_1_ t0, PIH_2_ t0 and PIH_3_ t0. (*n* = 8 horses, for all except the third induction where *n* = 7). Boxes depict median and interquartile range; whiskers denote minimum and maximum values.

In all joints, a clear inflammatory response was evident following each induction of LPS, seen as obvious peaks in the synovial total protein and synovial white blood cell counts (Figure 2a,b). Statistical methods were used to interrogate if each induction caused similar effects or if there was evidence of sensitisation or desensitization with repeated inductions. 

#### 3.1.1. Welfare Monitoring

Minimal changes were seen in the CWS across any of the inductions (Table A1). The highest score obtained was 4 in one horse at a single timepoint in the first induction, and all horses were back to their baseline scores of 0 by 24 h after each LPS induction. 

#### 3.1.2. Clinical Monitoring

Minimal changes were noted in joint effusion or joint flexion scores across any of the inductions (Table A1). 

**Figure 3:**
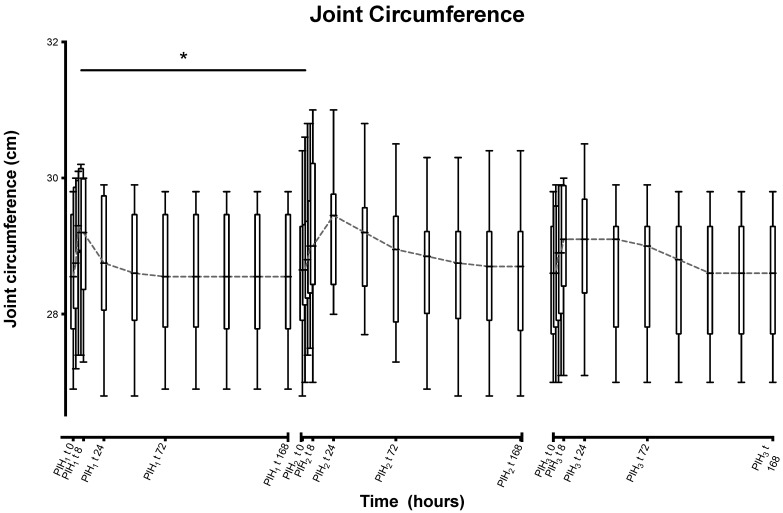
Joint circumference over time following repeated induction of inflammation with intra-articular injections of 0.25 ng of LPS in the middle carpal joint of horses at PIH_1_ t0, PIH_2 t_0 and PIH_3_ t0. (*n* = 8 horses, for all except the third induction where *n* = 7). Boxes depict median and interquartile range; whiskers denote minimum and maximum values. * *p* < 0.005 indicating where there are significant differences between timepoints.

Joint circumference showed a statistically significant increase between the peaks of the first and second inductions (0.587 cm, *p* = 0), but not between the second and third, or between the third and the first (Figure 3).

#### 3.1.3. Synovial Fluid Biomarker Monitoring (Table A2)

**Figure 4:**
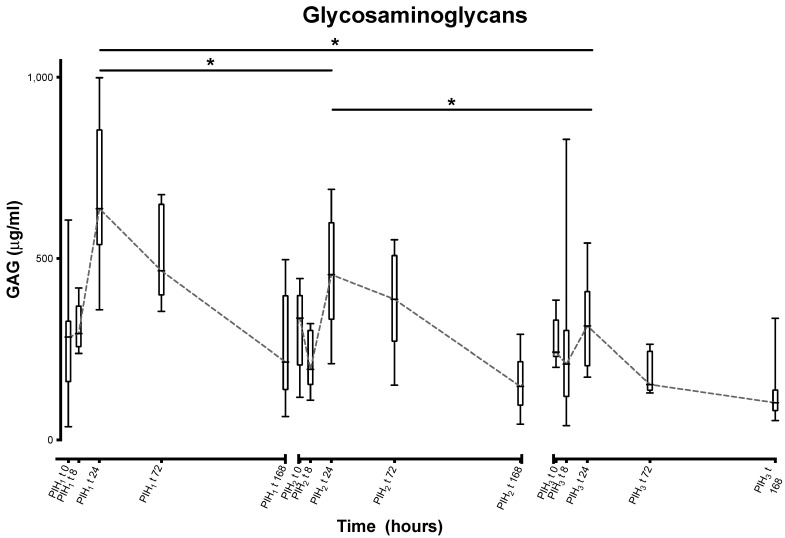
Synovial glycosaminoglycans over time following repeated induction of inflammation with intra-articular injections of 0.25 ng of LPS in the middle carpal joint of horses at PIH_1_ t0, PIH_2_ t0 and PIH_3_ t0. (*n* = 8 horses, for all except the third induction where *n* = 7). Boxes depict median and interquartile range; whiskers denote minimum and maximum values. * *p* < 0.005, indicating where there are significant differences between timepoints.

GAGs show statistically significant decreases in peak levels for all inductions (first to second—168.475 µg/mL, *p* = 0; second to third—181.461 ug/mL, *p* = 0), indicating a reasonably consistent decrease with each induction (Figure 4). 

**Figure 5:**
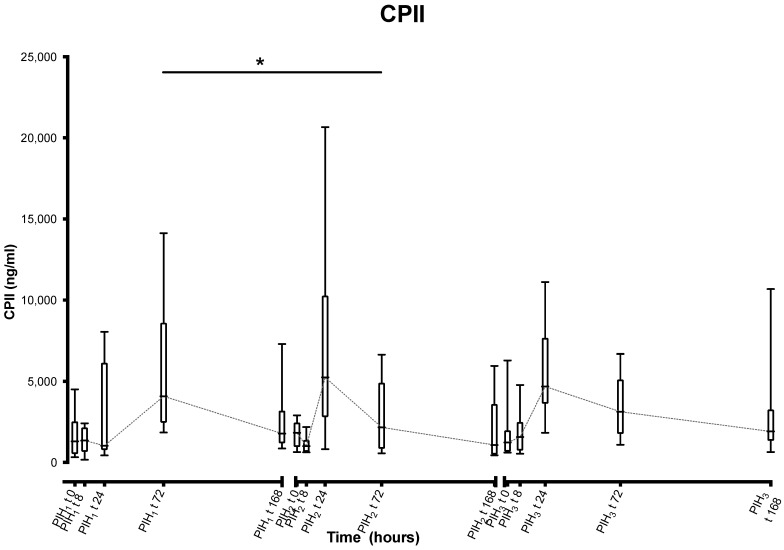
Synovial carboxypropeptide of type II collagen epitope (CPII) over time following repeated induction of inflammation with intra-articular injections of 0.25 ng of LPS in the middle carpal joint of horses at PIH_1_ t0, PIH_2_ t0 and PIH_3_ t0. (*n* = 8 horses, for all except the third induction where *n* = 7). Boxes depict median and interquartile range; whiskers denote minimum and maximum values. * *p* < 0.005, indicating where there are significant differences between timepoints.

CPII shows a statistically significant (−3075.425 ng/mL, *p* = 0.004) decrease between the levels found at the timepoint PIH_1_ t72 and timepoint PIH_2_ t72. It was planned to compare the levels at the timepoints 72 h post induction for this study, as a clear peak was noted at 72 h after the first induction (PIH_1_ t72). However, the visual inspection of the graphed data suggests that the peak appears to occur earlier after the second and third inductions, suggesting the planned contrasts may not have been the most appropriate for comparing “peak levels” (Figure 5). 

**Figure 6:**
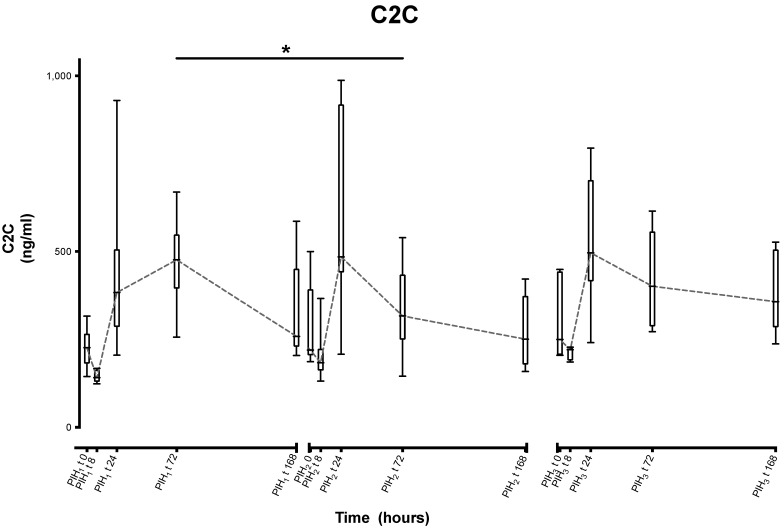
Synovial C2C over time following repeated induction of inflammation with intra-articular injections of 0.25 ng of LPS in the middle carpal joint of horses at PIH_1_ t0, PIH_2_ t0 and PIH_3_ t0. (*n* = 8 horses, for all except the third induction where *n* = 7). Boxes depict median and interquartile range; whiskers denote minimum and maximum values. * *p* < 0.005, indicating where there are significant differences between timepoints.

C2C similarly shows a statistically significant (−137.063 ng/mL, *p* = 0.002) decrease between the first and second peaks of the first and second induction, but as with CPII this difference would appear to be due to the difference in the timing of the peaks (Figure 6). 

**Figure 7:**
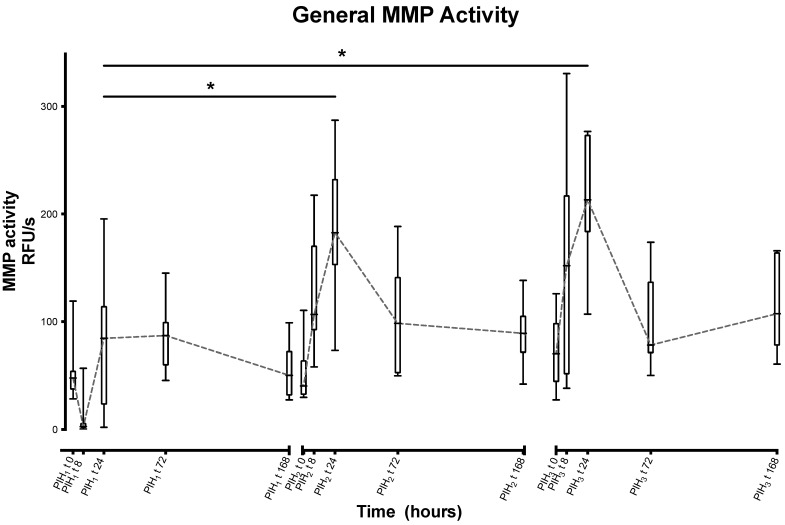
Synovial general matrix metalloproteinase activity over time following repeated induction of inflammation with intra-articular injections of 0.25 ng of LPS in the middle carpal joint of horses at PIH_1_ t0, PIH_2_ t0 and PIH_3_ t0. (*n* = 8 horses, for all except the third induction where *n* = 7). Boxes depict median and interquartile range; whiskers denote minimum and maximum values. * *p* < 0.005, indicating where there are significant differences between timepoints.

MMP showed a statistically significant increase between the peaks of the first and second inductions (103.8 RFU/s, *p* = 0), and the first and third (130.204 RFU/s, *p* = 0), but not between the second and third. Visual inspection of the graphed data here also suggests a difference in the evolution of the peaks when comparing the first to the second and third. (Figure 7). 

### 3.2. Research Question 2: Is There Evidence of Difference between Baseline (PIH_1_ t0) and Final Measurements?

#### 3.2.1. Welfare Monitoring

The composite welfare scores (CWS) showed no difference between the baseline (PIH_1_ t0) and the final measurements (Table A3).

#### 3.2.2. Clinical Monitoring

No evidence for a difference between baseline (PIH_1_ t0) and final measurements were found for joint effusion, joint flexion, or joint circumference. (Table A3). 

#### 3.2.3. Synovial Fluid Biomarker Monitoring 

For each of the synovial biomarkers measured, there was no significant difference found between the baseline and final measurements (Table A4). Inspection of the differences between the first and last timepoints indicated non-normality for WBCC, CCL2, and TNF-α, hence the Wilcoxon signed rank test was used as a non-parametric alternative to the paired *t*-test for these markers.

## 4. Discussion

With animal models of joint disease still clearly needed for the investigation of joint disease and therapeutics, a wide range of equine models are described in the literature. A comprehensive body of work has been done by the group at Colorado State University on a surgical post-traumatic osteoarthritis (PTOA) model [10,34,35] and, to a lesser degree, a chondral defect model [10,36]. Other groups have focused on inciting more generalized acute inflammation in the joint through the intra-articular administration of various synovitis-inducing substances such as amphotericin [37] or interleukin-1β [38]. Each model type has particular advantages and limitations, and the “perfect” model does not exist. The equine LPS model of intra-articular inflammation has become one of the most widely used for testing potential therapeutics [39,40]. With researchers becoming more cognizant of the need to reduce the harm to experimental animals, refinements of the model have been directed towards reducing the LPS dosage to a sub-nanogram level. De Grauw et al. [23] established that 0.5 ng of LPS elicited a marked, reliable, yet transient effect on certain synovial fluid biomarkers. However, the 0.5 ng dose of LPS was found to induce a relatively severe lameness in the equine subjects. Although this lameness was sufficiently transient and self-limiting, from an animal welfare viewpoint, it is still a significant harm. Particularly with the intention of modelling repeated inflammatory episodes, it was felt important to reduce the discomfort experienced by the horses during the inflammatory peaks even further in our model. 

In 1994, in one of the earliest studies utilizing intra-articular LPS in horses, it was established through dose-titration studies, that doses of LPS as low as 0.125 ng could induce a mild to moderate joint effusion, with a lower level of lameness [41]. However, a greater variability was seen in clinical signs, such as lameness, with doses lower than 0.5 ng. Two other studies by Meulyzer et al. [24] and Lucia et al. [28], used doses of 0.25 ng and 0.5 ng, respectively. In both studies, sizeable increases in synovial markers were found but in each only a limited number of biomarkers were investigated. With the intention of reducing welfare impact on the experimental animals, we chose to use the lower dose of LPS and focus on synovial biomarkers as outcomes measures, rather than the variable lameness seen with low-dose LPS studies. In the current study, we investigated an LPS dose of 0.25 ng and demonstrated reliable and marked responses across a large number of commonly reported synovial fluid biomarkers. Comparing the findings of this study with previous studies performed by our group using the 0.5 ng dose of LPS where the CWS were noted to be higher over a more prolonged period of time [42]. The clinical signs noted here are considerably less with the 0.25 ng dose, indicating reduced welfare impact overall with lower LPS dose.

The LPS model has been championed by our group and others due to its relatively low welfare impact, and the limited duration of the clinical effects on research animals can be considered a significant advantage of this model. However, this has also been cited as a significant limitation of the model in terms of its relevance to clinical disease, where chronic inflammation rather than acute self-limiting inflammation is recognized as the hallmark of naturally occurring OA [25,26,41]. A model that could mimic ongoing or repeated bouts of low-grade inflammation could be more indicative of natural disease state, and this study is a further step towards developing such a model. In one of the earlier LPS studies by Palmer and Bertone, repeated injections of 0.125 ng of LPS at 48 h intervals for four injections caused mild to moderate inflammation, more typical of clinical cases [41]. Kay et al. also used repeated injections of LPS in their study but used much higher doses of LPS (90–120 ng per joint) and repeated this at 5-day intervals for three injections in total [43]. Both studies demonstrated the potential to induce repeated episodes of inflammation but were limited in time. A more recent study by Cokelaere et al. expanded the timeframe towards three injections with 0.25 ng LPS at 14-day intervals but could not demonstrate consistent inflammation and discussed the potential of LPS tolerance [29]. 

The results of this investigation show that each LPS induction resulted in a reliable, marked inflammatory response reflected in our measured parameters. While Cokelaere et al. proposed the possibility of LPS tolerance, the findings of the present study would suggest that repeated LPS injections at 2-week intervals did not elicit signs of tolerance, while inflammatory symptoms remained similar over the different repeated inductions of inflammation. However, the extracellular matrix markers for GAGs and collagen (CPII, C2C) did show some variations between the subsequent inductions. For GAGs, significant decreases in peak synovial fluid concentrations were noted with repeated inductions. Increases in synovial fluid concentrations of GAGs have been previously documented in different equine models of joint disease [18,23] where induced inflammation or experimental injury to cartilage both led to increases in GAG release. Increased synovial fluid concentrations of GAGs have also been recognized in horses with clinical joint disease [18,44]. GAG loss from cartilage is recognized as a critical step in the pathogenesis of OA, eventually leading to physical changes in the cartilage that may predispose to cellular necrosis [45]. The reduction in response to insult seen in synovial fluid GAGs in this study could reflect a depletion of GAGs from the cartilage and suggests a reduced ability of the cartilage of the joint to recover following repeated insults. Similar pathogenesis was previously seen in an in vitro study using bovine cartilage explants, which demonstrated that, while recovery of GAG levels following IL-8-mediated depletion was possible, recovery was inversely proportional to the degree of insult [46]. GAG levels have been shown to have a rapid response to LPS stimulation [23], and it is possible that this fast response may have led to a transient depletion in response to repeated inductions within a relatively short (two week) time span. For C2C and CPII, more rapid responses were noted in the second and third inductions, with peak levels being observed at the timepoint 24 h post-induction, different from the first induction, where the peaks were seen at 72 h post-LPS induction. In previous work, we saw that C2C and CPII had similar, although less rapid, responses to LPS as GAGs [23]. The difference in responses noted in the second and third inductions here may be the result of the joints not being fully back to normal following the previous induction, with the change in timing of the peaks reflecting a cumulative effect of GAG breakdown or turnover. 

Both joint circumference and general MMP activity showed a significantly increased response to the second LPS induction compared to the first. Joint circumference was the only objective quantitative clinical measurement used in this study. General MMP activity is a sensitive indicator of synovial inflammation, so it is unsurprising that its patterns closely correlate with clinical signs. The increased and more rapid response seen with the second induction could indicate a sustained or aggregative inflammatory effect in the short term or an initial transient sensitization which did not persist in the third induction. 

LPS tolerance has been described in horses, and a study investigating the duration of systemic LPS tolerance in vivo in horses showed that signs of LPS tolerance lasted at least seven days but had waned by 14–21 days [47]. Based on the findings of the current study and the lack of tolerance seen in the studies of Palmer and Kay, it could be inferred that for local, intra-articular use of LPS, there is a reduced likelihood of tolerance at 14 days. Interestingly, reduced TNF-alpha response has been considered a hallmark of LPS tolerance [48] and in this study, while marked individual variation was seen in TNF-alpha values between horses, clear increases were seen with each induction of LPS. 

The second investigation of this study demonstrates that, based on clinical parameters and synovial fluid biomarker levels, no lasting effects remain, suggesting that this is a fully reversible model of repeated joint inflammation. This is important since scientifically sound preclinical large animal models are still essential for the investigation of high societal impact complex diseases with extremely limited treatment options, such as OA [12]. While it is well accepted that inflammation plays a pivotal role in the origin and progression of OA [3], finding appropriate models of intrasynovial inflammation remains challenging. Historic inflammatory models that provide a transient single inflammatory insult inherently lack the ability to provide good modelling for diseases that have recurrent inflammatory flares as an etiological hallmark. However, sustained inflammation in large animal models rightly raises ethical concerns. We believe that the presented preclinical model is a suitable compromise between model requirements and animal welfare considerations. The ability of the joints to recover fully from the repeated insults allows for the possibility of reuse or rehoming of the experimental animals, eliminating the need to sacrifice them. 

As with all experimental studies, there are a number of limitations to be acknowledged. While the repeated inductions of LPS led to inflammation being measurable over a prolonged period of time, the “peaks” and “troughs” seen with this model are still not completely representative of the natural disease processes, whereas with OA, for example, it would be expected to have a consistent, progressive low level of inflammation. However, in the absence of a perfect model, we believe that this longer duration model can provide a suitable testing platform for novel therapeutics compared to the single-severe insult inflammatory models currently used.

In addition, in this study we did not include any negative controls, which might have allowed comparison of the effect of the LPS with the effects of repeated arthrocentesis alone, which have been previously reported [16,17]. Therefore, it cannot be determined how much of the inflammatory responses seen can be attributed to the LPS and how much to the physical stimulation of the needle insertions and synovial fluid aspirations. However, comparing our results to those of a previous study where increases in markers of inflammation—synovial white blood cell counts and total protein—were seen in saline injected control joints [49], it is clear that those increases were substantially less than the increases noted here. Other studies have reported that significantly greater increases were seen in a number of synovial biomarkers, such as prostaglandin E2 and tumor necrosis factor-alpha, in LPS-injected joints compared to joints injected with a negative control (saline) [28,50]. Considering this clear evidence from earlier reports and the overarching aim to respect the principles of 3 R and reduce the number of animals used, we could not justify adding additional animals as controls.

## 5. Conclusions

It can be concluded that the lower dose of 0.25 ng LPS gives a reliable intra-articular inflammatory response and leads to a low level of discomfort for the experimental animals. Repeated LPS-induced inflammation could be produced with no evidence of sustained LPS tolerance or sensitization and still allows for complete recovery of the joints following the interventions. Furthermore, the consistent, reliable repeated intra-articular inflammatory response produced across a panel of biomarkers with repeated injections of 0.25 ng LPS suggests potential for the effects of interventions or novel therapeutics to be investigated in a more prolonged model of recurrent joint inflammation.

## Data Availability

The data are not in a formal data management store; however, the authors can be contacted for the dataset to be sent as requested.

## Appendix A. Composite Welfare Score Sheet for the Equine LPS Model

**Parameter**	**Animal ID**	**Score**	**Date/Time**
Food and water intake	Normal	0	
	Moderate	1	
	Low	2	
	No food or water intake	4	
Clinical parameters	Normal temperature (T), cardiac (C) and respiratory (R)rates	0	
	Slight changes	1	
	T ± 1 °C, C/R rates increase more than 30%	2	
	T ± 2 °C, C/R rates increase more than 50%	4	
Natural behaviour	Normal	0	
	Minor Changes	1	
	Less mobile and alert	2	
	Restless or still	4	
Provoked behaviour	Normal	0	
	Minor depression or exaggerated response	1	
	Moderate change in expected behaviour	2	
	Reacts violently, or very weak and pre-comatose	4	
	Total	0–16	
**Score**	**Action**		
0–3	Normal, no action to be taken		
4–8	Monitor carefully, consider analgesics		
9–12	Seek second opinion from named animal care and welfare officer and/or named veterinary surgeon. Consider euthanasia.		
13–16	Indicates severe pain. Seek immediate second opinion from named veterinary surgeon. Animal withdrawn from project. Based on advice from named veterinary surgeon, initiate appropriate treatment and analgesia. If animal’s symptoms cannot be alleviated, again in consultation with the named veterinary surgeon, consider euthanasia.		

Table A1: Welfare and clinical scores across 3 LPS inductions. Composite welfare score (CWS) is the aggregate of scores (scale 0–4) for each of the categories as described in Appendix A: food and water intake; clinical parameters; natural behavior; and provoked behavior, over time following repeated induction of inflammation with intra-articular injections of 0.25 ng of LPS in the middle carpal joint of horses at PIH_1_ t0, PIH_2_ t0 and PIH_3_ t0. Data correspond to the mode (*n* = 8 joints for each treatment group, except for the third induction where *n* = 7 for each group). Joint effusion is a score (scale 0–4) for observed/palpated joint effusion over time following repeated induction of inflammation with intra-articular injections of 0.25 ng of LPS in the middle carpal joint of horses at PIH_1_ t0, PIH_2_ t0 and PIH_3_ t0. Data correspond to the mode (*n* = 8 joints for each treatment group, except for the third induction where *n* = 7 for each group). Joint flexion is a score (scale 0–4) to subjectively grade the horse’s response to passive flexion of the carpus, where 0 indicates the horse has no reaction and 4 indicates that the horse completely resists any attempt to flex the carpus. Data correspond to the mode (*n* = 8 joints for each treatment group, except for the third induction where *n* = 7 for each group). Joint circumference is the measurement in cm of the carpus at a fixed (marked) point over time following repeated induction of inflammation with intra-articular injections of 0.25 ng of LPS in the middle carpal joint of horses at PIH_1_ t0, PIH_2_ t0 and PIH_3_ t0. Data correspond to the mean ± standard deviation of the mean (*n* = 8 horses, for all except the third induction where *n* = 7).

**Table A1:** Welfare and Clinical Scores Across 3 LPS Inductions.

	LPS Induction	Timepoint											
		0	2	4	6	8	24	48	72	96	120	144	168
CWS	PIH_1_	0	0	0	0.5	0	0	0	0	0	0	0	0
	PIH_2_	0	0	0	0	0	0	0	0	0	0	0	0
	PIH_3_	0	0	0	0	0	0	0	0	0	0	0	0
Joint Effusion Score	PIH_1_	0	0	1	1	1	0	0	0	0	0	0	0
	PIH_2_	0	0	0	0	0	0	0	0	0	0	0	0
	PIH_3_	0	0	0	0	1	1	0	0	0	0	0	0
Joint Flexion Score	PIH_1_	0	0	0	0	0	0	0	0	0	0	0	0
	PIH_2_	0	0	0	0	0	0	0	0	0	0	0	0
	PIH_3_	0	0	0	0	0	0	0	0	0	0	0	0
Joint Circumference (cm)	PIH_1_	28.55 ± 1.00	28.84 ± 1.01	29.23 ± 0.88	29.26 ± 0.93	29.09 ± 0.98	28.73 ± 1.06	28.60 ± 1.01	28.56 ± 0.99	28.56 ± 0.99	28.55 ± 1.00	28.55 ± 1.00	28.55 ± 1.00
	PIH_2_	28.64 ± 1.08	28.74 ± 1.05	28.90 ± 1.00	20.03 ± 1.00	20.18 ± 1.25	29.31 ± 0.95	29.13 ± 0.93	28.85 ± 1.03	28.70 ± 1.01	28.65 ± 1.01	28.64 ± 1.07	28.61 ± 1.09
	PIH_3_	28.50 ± 0.93	28.57 ± 0.99	28.64± 0.98	28.80 ± 1.00	28.93 ± 0.98	29.07 ± 1.10	28.71 ± 1.00	28.69 ± 0.99	28.59 ± 0.95	28.51 ± 0.94	28.50 ± 0.93	28.50 ± 0.93

Table A2: Synovial fluid analysis across 3 LPS inductions Comparison of synovial fluid total protein (TP), white blood cell count (WBCC), prostaglandin E_2_ (PGE_2_) bradykinin, CCL2, tumor necrosis factor-α (TNF-α), general matrix metalloproteinase activity (MMP), glycosaminoglycans (GAG), collagen-cleavage neoepitope of type II collagen (C2C) and carboxypropeptide of type II collagen epitope (CPII) over time following repeated induction of inflammation with intra-articular injections of 0.25 ng of LPS in the middle carpal joint of horses at PIH_1_ t0, PIH_2_ t0 and PIH_3_ t0. (*n* = 8 horses, for all except the third induction where *n* = 7). Data correspond to the mean ± standard deviation of the mean for all parameters.

**Table A2:** Synovial Fluid Analysis Across 3 LPS Inductions.

	LPS Induction	Timepoint				
		0	8	24	72	168
Total Protein (g/L)	PIH_1_	19.00 ± 4.00	56.25 ± 16.12	44.75 ± 13.13	22.50 ± 5.93	18.75 ± 4.27
	PIH_2_	21.00 ± 2.39	48.75 ± 16. 07	50.50 ± 7.31	22.50 ± 6.48	25.75 ± 6.63
	PIH_3_	22.57 ± 2.76	47.71 ± 14.21	47.14 ± 3.02	25.71 ± 2.14	28.29 ± 5.09
WBCC Cells × 10^9^/L	PIH_1_	0.07 ± 0.03	102.83 ± 39.57	32.21 ± 41.27	1.99 ± 2.56	0.20 ± 0.14
	PIH_2_	0.08 ± 0.38	95.4 ± 61.49	39.88 ± 23.13	1.13 ± 1.27	0.16 ± 0.05
	PIH_3_	0.09 ± 0.08	84.32 ± 68.12	20.40 ± 14.34	0.75 ± 1.05	0.08 ± 0.12
PGE_2_ (pg/mL)	PIH_1_	18.69 ± 9.21	1336.03 ± 1201.01	56.82 ± 35.79	27.64 ± 13.41	29.95 ± 21.70
	PIH_2_	21.24 ± 16.78	2034.24 ± 3585.06	151.50 ± 140.98	29.81 ± 11.66	45.40 ± 46.22
	PIH_3_	23.72 ± 19.69	1173.65 ± 1196.42	65.90 ± 85.15	350.98 ± 832.11	16.67 ± 12.10
Bradykinin (ng/mL)	PIH_1_	47.95 ± 19.36	171.39 ± 154.97	91.83 ± 47.42	50.25 ± 19.44	42.68 ± 18.81
	PIH_2_	32.69 ± 15.55	152.68 ± 123.53	98.23 ± 59.60	65.58 ± 31.96	62.64 ± 28.20
	PIH_3_	41.61 ± 10.45	143.49 ± 130.08	156.73 ± 20.33	76.34 ± 23.51	78.69 ± 23.15
CCL2 (pg/mL)	PIH_1_	203.50 ± 169.98	2293.67 ± 1139.77	1185.68 ± 889.45	246.34 ± 166.02	146.25 ± 130.92
	PIH_2_	124.00 ± 103.65	15,185.69 ± 19,325.94	402.37 ± 226.77	229.62 ± 159.45	198.66 ± 130.13
	PIH_3_	156.71 ± 114.92	15,731.97 ± 24,035.09	337.03 ± 273.05	223.14 ± 199.74	283.43 ± 285.06
TNF-α (pg/mL)	PIH_1_	0.00 ± 0.00	196.26 ± 217.00	28.48 ± 47.17	0.96 ± 2.72	0.94 ± 2.65
	PIH_2_	0.00 ± 0.00	138.48 ± 163.90	7.8 0± 14.05	1.83 ± 5.16	1.24 ± 3.50
	PIH_3_	1.61 ± 4.27	209.46 ± 305.63	5.16 ± 6.79	3.70 ± 9.06	6.26 ±12.93
MMP (RFU/s)	PIH_1_	53.75 ± 27.93	10.74 ± 20.49	81.53 ± 61.62	83.38 ± 31.15	54.98 ±24.59
	PIH_2_	51.53 ± 27.26	126.84 ± 52.95	185.33 ± 65.41	102.76 ± 53.12	88.41 ± 28.60
	PIH_3_	72.11 ± 34.69	157.96 ± 104.59	211.73 ± 59.89	99.61 ± 43.99	115.56 ± 45.17
GAG (µ/mL)	PIH_1_	311.30 ± 154.79	298.95 ± 48.88	660.31 ± 188.59	508.65 ± 129.52	244.76 ± 152.21
	PIH_2_	335.82 ± 86.17	227.28 ± 94.47	491.82 ± 162.68	382.71 ± 134.17	147.37 ± 64.18
	PIH_3_	292.32 ± 76.71	201.11 ± 121.04	318.39 ± 124.69	173.69 ± 51.74	184.00 ± 173.84
C2C (ng/mL)	PIH_1_	225.39 ± 54.80	145.23 ± 17.13	438.89 ± 223.96	471.03 ± 124.79	323.13 ± 141.09
	PIH_2_	290.06 ± 119.48	204.18 ± 71.93	597.20 ± 278.05	333.96 ± 127.99	271.29 ± 100.51
	PIH_3_	314.4 ± 112.70	209.87 ± 18.43	545.51 ± 195.05	411.79 ± 138.86	376.06 ± 108.84
CPII (ng/mL)	PIH_1_	1645.54 ± 1406.82	1354.94 ± 793.56	2746.41 ± 3128.26	5783.03 ± 4286.34	2493.85± 2106.06
	PIH_2_	1741.20 ± 811.48	1097.99 ± 527.90	7259.01± 6356.18	2707.60 ± 2270.28	2034.06 ± 2031.00
	PIH_3_	1951.24 ± 1978.21	1880.30 ± 1471.47	5565.04 ± 3013.79	3328.23 ± 2002.97	3219.21 ± 3409.41

Table A3: Welfare and clinical scores baseline vs. final timepoint: Composite welfare score (CWS) is the aggregate of scores (scale 0–4) for each of the following categories: food and water intake; clinical parameters; natural behavior; and provoked behavior, with the baseline value being prior to any induction of inflammation with LPS (PIH1 t0) and the final sampling timepoint, 9 weeks after the last induction. Data correspond to the mode (*n* = 8 joints for each group). Joint Effusion is a score (scale 0–4) for observed/palpated joint effusion, with the baseline value being prior to any induction of inflammation with LPS (PIH_1_ t0) and the final sampling timepoint, 9 weeks after the last induction. Data correspond to the mode (*n* = 8 joints for each group). Joint Flexion is a score (scale 0–4) to subjectively grade the horse’s response to passive flexion of the carpus, where 0 indicates the horse has no reaction and 4 indicates that the horse completely resists any attempt to flex the carpus, with the baseline value being prior to any induction of inflammation with LPS (PIH_1_ t0) and the final sampling timepoint, 9 weeks after the last induction. Data correspond to the mode (*n* = 8 joints for each group). Joint circumference is the measurement in cm of the carpus at a fixed (marked) point, with the baseline value being prior to any induction of inflammation with LPS (PIH1 t0) and the final sampling timepoint, 9 weeks after the last induction. Data correspond to the mean ± standard deviation of the mean (*n* = 8 horses).

**Table A3:** Welfare and Clinical Scores Baseline vs. Final Timepoint.

	Timepoint	
	Baseline PIH_1_ t0	Final Timepoint
CWS	0	0
Joint Effusion Score	0	0
Joint Flexion Score	0	0
Joint Circumference (cm)	28.6 ± 1	28.63 ± 0.91

Table A4: Synovial fluid analysis baseline vs. final timepoint: Comparison of synovial fluid total protein (TP), white blood cell count (WBCC), prostaglandin E_2_ (PGE_2_) bradykinin, CCL2, tumor necrosis factor-α (TNF-α), general matrix metalloproteinase activity (MMP), glycosaminoglycans (GAG), collagen-cleavage neoepitope of type II collagen (C2C) and carboxypropeptide of type II collagen epitope (CPII) with the baseline value being prior to any induction of inflammation with LPS (PIH_1_ t0) and the final sampling timepoint, 9 weeks after the last induction. Data correspond to the mean ± standard deviation of the mean (*n* = 8 horses).

**Table A4:** Synovial Fluid Analysis Baseline vs. Final Timepoint.

	Timepoint	
	Baseline PIH_1_ t0	Final Timepoint
Total Protein (g/L)	19.00 ± 4.00	18.25 ± 5.82
WBCC Cells × 10^9^/L	0.07 ± 0.03	0.03 ± 0.05
PGE_2_ (pg/mL)	18.69 ± 9.21	27.44 ± 9.36
CCL2 (pg/mL)	203.5 ± 169.98	120.5 ± 123.39
TNF-α (pg/mL)	0.00 ± 0.00	0.31 ± 0.88
MMP (RFU/s)	53.75 ± 27.93	39.29 ± 18.65
GAG (µ/mL)	311.3 ± 154.79	251.33 ± 67.72
C2C (ng/mL)	225.39 ± 54.80	214.51 ± 71.46
CPII (ng/mL)	1645.54 ± 1406.82	1790.23 ± 2192.11
